# COVID-19 vaccination and antirheumatic therapy

**DOI:** 10.1093/rheumatology/keab223

**Published:** 2021-03-12

**Authors:** Jack Arnold, Kevin Winthrop, Paul Emery

**Affiliations:** 1 Leeds Institute of Rheumatic and Musculoskeletal Medicine, University of Leeds, Chapel Allerton Hospital, Leeds, UK; 2 Division of Infectious Diseases, Oregon Health and Science University, Portland, OR, USA; 3 NIHR Leeds Biomedical Research Centre, Leeds Teaching Hospitals NHS Trust, Leeds, UK

**Keywords:** COVID-19, vaccine, biologics, DMARDs, rituximab, methotrexate

## Abstract

The coronavirus disease 2019 (COVID-19) vaccination will be the largest vaccination programme in the history of the NHS. Patients on immunosuppressive therapy will be among the earliest to be vaccinated. Some evidence indicates immunosuppressive therapy inhibits humoral response to the influenza, pneumococcal and hepatitis B vaccines. The degree to which this will translate to impaired COVID-19 vaccine responses is unclear. Other evidence suggests withholding MTX for 2 weeks post-vaccination may improve responses. Rituximab has been shown to impair humoral responses for 6 months or longer post-administration. Decisions on withholding or interrupting immunosuppressive therapy around COVID-19 vaccination will need to be made prior to the availability of data on specific COVID-19 vaccine response in these patients. With this in mind, this article outlines the existing data on the effect of antirheumatic therapy on vaccine responses in patients with inflammatory arthritis and formulates a possible pragmatic management strategy for COVID-19 vaccination.

**Rheumatology key messages**

- Existing work on vaccine response in DMARDs is an imperfect surrogate for COVID-19 vaccine response.
- MTX may impair humoral response; rituximab likely impairs humoral response for 6 months or longer.
- Consider risk stratifying rituximab-treated patients and delaying/postponing therapy if appropriate before COVID-19 vaccination.

## Introduction

The aim of this viewpoint article is to outline the existing data on the effect of antirheumatic therapy on vaccine responses in patients with inflammatory arthritis, and to formulate a possible pragmatic strategy for the management of therapies in these patients in the context of prospective coronavirus disease 2019 (COVID-19) vaccination. But primarily we aim to facilitate an informed discussion between clinicians and patients in response to issues raised by these data.

COVID-19 needs little introduction, and the three effective vaccines produced by Pfizer (mRNA vector), Moderna (mRNA vector) and AstraZeneca (chimpanzee adenovirus vector ChAdOx-1) have provided a potential exit strategy. The COVID-19 vaccine rollout will be the largest mass vaccination programme in the history of the NHS.

Vaccinations exert their protective effect by stimulating both humoral and cellular immune responses. The relative importance of humoral and cellular immunity in conferring protection from infection varies with each infective organism [1]. B-cell responses are better represented in the literature due to their ease of antibody measurement and the lack of a clear immune correlate of protection for T-cell driven responses. Nevertheless, it is worth noting that emerging evidence suggests a strong role for T-cell mediated immunity in COVID-19 infection [2, 3].

Immunosuppressive therapy such as the DMARDs, used to treat most of our patients, may impair vaccine responses. Existing data on this topic largely focus on influenza, pneumococcal and tetanus vaccines. There is a small amount of data also available on the Zostavax vaccine. Whether these data can be extrapolated to provide guidance for vaccination strategies in COVID-19 remains uncertain. Patients on immunosuppressive therapy are being prioritized for vaccination, so management decisions will need to be made prior to any additional COVID-19 data being available. Caveats when assessing the literature are noted below in Table 1.

**Table 1:** Outline of key caveats when assessing the existing literature on vaccine response in the context of DMARD/biologic therapies


Most literature focuses on influenza or pneumococcal vaccine responses, with a smaller number of studies evaluating tetanus/HBV vaccines. The validity of extrapolating these studies to COVID-19 vaccination using novel (e.g. mRNA) vaccine platforms is uncertain.
Most studies assess only humoral responses to vaccination with limited data on T-cell responses, which may be more important in conferring viral immunity.
The degree to which reduced antibody titres on vaccination correlate with impairment of immunity to COVID-19 is uncertain. T-cell mediated immunity is likely to be important in COVID-19 vaccination based on data to date. However, currently there is no known immune correlate of protection identified.

## Impact of antirheumatic therapy on vaccine response

Table 2 below summarizes a review of the literature on the impact of anti-rheumatic therapy on vaccine response. Further discussion is under the relevant headings.

**Table 2:** Summary of the evidence for the effect of common DMARDs/biologic therapies on vaccine response

Drug	Findings	Interpretation/advice on management
Corticosteroids	Doses >10 mg prednisolone daily associated with impaired humoral immunity [1–5]. Doses <10 mg prednisolone daily not shown to impair humoral response. Doses >10 mg prednisolone daily associated with poorer outcomes in hospitalized COVID-19 patients [6].	Channelling bias present, as patients on steroid therapy generally sicker. Could consider tapering prednisolone to <10 mg daily where possible, likely already standard practice.
csDMARDs (not MTX)	Small reduction in vaccine-induced antibody levels but maintained seroprotective titres [7–11]. Most MMF data from transplant patients [11].	Continue therapy.
MTX (alone or in combination)	MTX may impair humoral response to pneumococcal and influenza vaccines [12–14]. Limited data suggesting an improved humoral response to influenza vaccine if MTX held for 2 weeks post-vaccination [13]. Withholding for >2 weeks associated with increased flare risk without further improvement in vaccine response [13].	Some evidence to hold for 2 weeks post-vaccination. Limited generalizability and may increase flare risk. Need further data.
Anti-TNF	Quantitative but not significant impairment of humoral vaccine response with influenza [1, 5, 7, 15] but limited evaluation. Some impairment of response to HBV vaccine demonstrated [16, 17].	Continue therapy.
Anti-IL-6	No significant impairment of humoral vaccine response [18, 19].	Continue therapy.
Abatacept	Conflicting data, shown to impair influenza vaccine response in 2011 [20]. Normal results for S/C preparation assessing influenza and pneumococcal vaccine response [21]. Small study showed impairment of PCV-7 response [22].	Conflicting evidence but no clear evidence to discontinue. Limited evaluation of data, no control group on two studies [20, 21]. Only 17 abatacept-treated patients in other study [22]. Need further data especially on theoretical effect on T-cell responses.
JAK inhibitors	Baricitinib-treated patients shown to mount effective PCV-13 vaccine response, but less robust tetanus responses [23]. T-cell responses to the PCV-13 vaccine were preserved in tofacitinib-treated patients [24]. Tofacitinib did reduce influenza vaccine titres but seroprotective titres were preserved [25]. cPPSV-23 seroprotective responses were lower than placebo controls [25]. Among present tofacinitib users, discontinuing tofacitinib for 1 week before and after vaccination had no effect upon the proportion of patients reaching seroprotection [25]. Tofacitinib shown to be safe in context of live zoster vaccine, starting tofacitinib 2–3 weeks post-vaccination yielded similar humoral and cell-mediated responses to controls [26].	Evidence suggests some diminished humoral responses to influenza and PPSV-23. Biologically plausible that may inhibit mRNA vaccines with a substantial interferon driven response.
Anti-CD20	Shown to impair humoral response to both PPSV-23 and influenza vaccine [3, 12, 27, 28]. Effect most pronounced if vaccinated earlier <3 months after rituximab therapy [27, 28]. Improved vaccine response if vaccinated >6 months after RTX therapy [27, 28].	Aim to vaccinate before RTX or >6 months post-RTX treatment where possible. Could consider postponed therapy in specific cases.

csDMARD: conventional synthetic DMARD; JAK: Janus kinase; PCV-7: heptavalent pneumococcal conjugate vaccine; PCV-13: 13 valent pneumococcal conjugate vaccine; PPSV-23: pneumococcal polysaccharide vaccine; RTX: Rituximab; S/C: Subcutaneous injection.

### Corticosteroids

Corticosteroids affect vaccine efficacy in a dose-dependent manner. Several studies have assessed the impact of corticosteroid therapy on humoral response to the pneumococcal and influenza vaccines [4–8]. Doses >10 mg prednisolone daily were associated with a degree of impaired humoral immunity in a longitudinal study; however, lower doses had little impact [5]. Steroid doses >10 mg daily prednisolone were associated with poorer outcomes in hospitalized patients with COVID-19[9]. 

### csDMARDs

Other than MTX, there is limited evidence for significant impairment of humoral vaccine responses to other conventional synthetic DMARDs (csDMARDs). Sulfasalazine, hydroxychloroquine, azathioprine and leflunomide may reduce vaccine antibody titres but have not been shown to inhibit a seroprotective response to the pneumococcal or influenza vaccines [10–14].

Much of the trial data on mycophenolate is from organ transplant patients. These trials did not assess responses where mycophenolate was withheld, due to the high risk of graft rejection [14]. Mycophenolate was shown to reduce antibody titres but not below the threshold for seroprotection.

MTX has been shown to impair humoral response to the pneumococcal and influenza vaccines [15–18]. This is unsurprising given its ability (and use) to reduce antibody formation to monoclonal antibodies. Withholding MTX around the time of vaccination has been assessed for 4 weeks before influenza vaccination, 2 weeks either side of vaccination and 4 weeks post-vaccination [16, 18]. Holding MTX for 4 weeks after immunization substantially improved vaccine titres. A subsequent study suggested that the critical period for vaccine-induced humoral immunity was the 2-week period following vaccination [16, 18]. Longer periods of withholding MTX were not shown to confer better vaccine responses but were associated with an increased incidence of disease flare. It appears MTX has the same impact on vaccination when used in combination with other DMARDs. The same risk–benefit assessment is required for a decision on temporary withholding.

### TNF inhibition

Several studies have assessed the impact of anti-TNF therapies on pneumococcal and influenza vaccines. There have been no consistent data linking these treatments to significant impairment of the immune response. However, in patients who are taking concurrent MTX, responses have been shown to be impaired. While seroprotective responses are typically maintained, vaccine antibody titres may be lower than for matched controls [4, 8, 10, 19]. TNF inhibition has also been shown to be safe in the context of the live varicella zoster vaccine [20]. In the context of COVID-19, early registry data have shown anti-TNF therapy to be associated with a decreased odds of hospitalization due to COVID-19 [9].

### IL-6 inhibitors

Two large Japanese studies have assessed the impact of IL-6 inhibition on influenza and pneumococcal vaccine responses. One showed impaired responses in the IL-6 inhibitor plus MTX combination treatment arm but no impairment with IL-6 inhibition monotherapy [21]. A subsequent study showed no significant impairment in humoral response to the influenza and pneumococcal vaccines at 12 weeks in tocilizumab-treated patients [22].

### Abatacept

There is some conflict within the existing data. Abatacept was shown to impair response to the H1N1 influenza vaccine in comparison to age-matched patients [23]. Abatacept was shown to impair heptavalent pneumococcal conjugate vaccine (PCV-7) responses in another small volume study with 17 abatacept-treated patients enrolled [24]. However, subsequent work showed no impairment of response to the trivalent influenza and pneumococcal polysaccharide vaccines in patients treated with subcutaneous abatacept at a dose of 125 mg weekly [25]. Interpretation of data is problematic as the two papers lacked a control group and one study recruited only 17 abatacept-treated patients.

### Janus kinase inhibitors

Janus kinase (JAK) inhibition may prove to be problematic in the context of the mRNA COVID-19 vaccines, which induce a strong type 1 interferon-driven immune response. Theoretically, inhibition of this pathway could be associated with a diminished response.

The effect of baricitinib on pneumococcal conjugate and tetanus toxoid vaccine response was assessed and showed that 68% of patients on long-term baricitinib mounted seroprotective responses to the pneumococcal vaccine, although tetanus toxoid responses were less durable [26].

One study assessed the impact of tofacitinib (plus MTX in half of cases) on pneumococcal polysaccharide vaccine (PPSV-23) and influenza vaccine response [27]. Here similar proportions of tofacitinib and control patients achieved a satisfactory response to the influenza vaccine, but pneumococcal responses were impaired, particularly when tofacitinib was combined with MTX. Temporary discontinuation of tofacitinib therapy for 1 week pre-vaccination until 1 week after vaccination was not shown to impact on the humoral response.

Recent data in an abstract from the ACR Convergence 2020 has suggested a satisfactory response to the adjuvant herpes subunit zoster vaccine in JAK-inhibitor-treated patients [28]. However, one-quarter of the JAK-inhibitor-treated patients failed to mount any humoral vaccine response at all. Additionally, the live zoster vaccine Zostavax has been shown to be safe and effective in tofacitinib-treated patients in a study where similar VZV-specific humoral and cell-mediated responses were seen in controls and patients who started tofacitinib 2–3 weeks after live zoster vaccine administration [29].

### Anti-CD20

B-cell depleting therapy has been shown to impair humoral responses to the influenza and pneumococcal vaccines in several studies and a subsequent meta-analysis [6, 15, 30, 31]. Biologically, this is consistent with the critical role of B cells in humoral vaccine responses. In 2008, csDMARD-treated patients were compared with csDMARD/rituximab combination therapy patients in the context of the influenza vaccine. Lower antibody titres were identified to all antigens in the combination therapy group and were statistically significant in one case [6]. One study assessed influenza vaccine response in early (4–8 weeks) and late (6–10 months) rituximab-treated patients [31]. Impairment of response was greater in the early rituximab treatment arm. Another study showed general impairment of humoral responses to the influence vaccine after rituximab therapy, but better humoral responses in the late (>5 months post-treatment) rather than early treatment groups [30].

There are some early data suggesting worse outcomes particularly in rituximab treated COVID-19 patients. Case reports have described severe COVID-19 phenotypes in patients treated with rituximab for rheumatological and other B-cell driven disorders [32–34]. Early study data have in some cases suggested poorer outcomes in rituximab-treated patients who become hospitalized with COVID-19 [35, 36]. However, it is likely there is a significant channelling bias as rituximab-treated patients generally have higher rates of interstitial lung disease and other factors associated with poorer outcomes in COVID-19. Nevertheless, such data are concerning and reinforce the need for judicious use of rituximab for only the most clinically necessary cases during a global pandemic.

## Risk stratifying and timing vaccinations

Rheumatology departments require guidance on how to manage DMARD/biologic therapies in the context of mass COVID-19 vaccination and this guidance will evolve with time. Existing EULAR guidance is available but may not be sufficient in the context of a global pandemic [37]. In every case the benefits of reducing medication needs to be weighed against the risk of disease flare, which apart from the obvious disadvantage is known to reduce vaccination effectiveness [38]. Key considerations are summarized in Table 3 below.

**Table 3:** Outline of key therapeutic considerations when vaccinating against COVID-19 alongside DMARDs/biologic therapies

Where appropriate: Avoid vaccination during disease flare. Taper steroid therapy to <10 mg prednisolone daily. Consider withholding MTX for 2 weeks post-vaccination both when used as monotherapy and in combination with other DMARDs. (As two doses of current vaccines are required, this may would need to be done twice). Avoid vaccinating ideally for 6 months post-rituximab; if vaccination is imminent consider delaying rituximab infusion if no risk of organ failure/disease flare. If a patient is unlikely to receive vaccination for 6 months there is an argument for expediting RTX treatment. If there is insufficient time to alter or amend DMARD/biologic treatment, then we would recommend vaccination and reassessment of vaccine response at a later date.

**Fig. 1:**
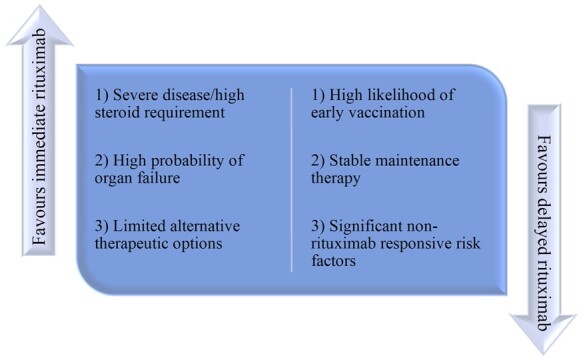
Factors influencing rituximab treatment decisions

A summary of the possible challenges specific to rituximab is depicted in Fig. 1.

For MTX, withholding treatment for 2 weeks following each vaccine dose may help improve humoral response. This is speculative in the context of novel vaccine techniques but could be considered in patients on MTX (and perhaps JAK inhibitors) at low to moderate risk of disease flare. Where a flare occurs, they would require treatment and high doses of prednisolone should be avoided where possible due to its possible effects on vaccine responses and COVID-19 morbidity. However, once again, it is important to stress that the priority is to proceed with vaccination and modification of therapy should not delay this.

The situation with JAK inhibitors is unknown, as unlike the MTX study they have only been withheld for 1 week post-vaccination so far. Some data from work on the influenza and PPSV-23 vaccines and the strong type 1 interferon response generated by the mRNA vaccines suggest that withholding JAK inhibitors might improve COVID-19 vaccine responses, but this is speculative. While for abatacept, the data are conflicting and given its mode of action, which could inhibit T-cell responses, treatment guidance urgently requires further evidence.

In all cases, any decision to delay treatment should be the result of an informed discussion by each patient and physician on a case-by-case basis.

## Further work

Additional COVID-19-specific data will be critical in producing more evidence-based recommendations. Quantification of COVID-19 vaccine antibody titres, evidence on T-cell immunity and additional work on the impact of booster vaccinations will all be relevant, and there is ongoing work in Leeds collecting such data with and without medication modification. COVID-19 is likely to be a long-term issue, and the data from such a study should be of value for advice on protection and optimal future vaccination strategy.
